# Novel set of plasma proteins classifies Alzheimer's dementia in African Americans with high accuracy

**DOI:** 10.1002/alz70856_100297

**Published:** 2025-12-25

**Authors:** Lindsey A. Kuchenbecker, Kevin J. Thompson, Cheyenne D. Hurst, Yen‐Ning Huang, Bianca M. Opdenbosch, Michael G. Heckman, Joseph S. Reddy, Thuy T. Nguyen, Heidi L. Casellas, Katie D. Sotelo, Delila J. Reddy, Wei Tsai, John A. Lucas, Gregory S Day, Floyd B. Willis, Vijay K. Ramanan, Neill R. Graff‐Radford, Nilüfer Ertekin‐Taner, Kwangsik Nho, Krishna Rani Kalari, Minerva M. Carrasquillo

**Affiliations:** ^1^ Mayo Clinic, Jacksonville, FL, USA; ^2^ Mayo Clinic, Rochester, MN, USA; ^3^ Center for Neuroimaging, Department of Radiology and Imaging Sciences, Indiana University School of Medicine, Indianapolis, IN, USA; ^4^ Indiana Alzheimer's Disease Research Center, Indiana University School of Medicine, Indianapolis, IN, USA; ^5^ Mayo Clinic in Florida, Jacksonville, FL, USA; ^6^ Indiana Alzheimer Disease Research Center, Indiana University School of Medicine, Indianapolis, IN, USA; ^7^ Center for Neuroimaging, Department of Radiology and Imaging Sciences, Indiana University School of Medicine, Indianapolis, IN, USA; ^8^ Indiana University School of Informatics and Computing, Indianpolis, IN, USA

## Abstract

**Background:**

Plasma biomarkers offer improved accessibility compared to established diagnostic modalities for Alzheimer's disease (AD). However, current plasma biomarker candidates have not been tested comprehensively in diverse populations, and represent only a fraction of pathways dysregulated in AD. This study seeks to address these knowledge gaps through untargeted plasma biomarker discovery in African Americans (AA), an underrepresented group in AD research despite having a higher risk of developing dementia compared to non‐Hispanic Whites (NHW).

**Method:**

Untargeted proteomics were generated using the SomaScan 7k platform on plasma samples from AA study participants clinically diagnosed as AD dementia (*n* = 181) or cognitively unimpaired (CU; *n* = 142). Machine learning was implemented to identify a set of plasma proteins to distinguish between AD dementia and CU. Predictive models were constructed by elastic net regression optimized with nested cross‐validation on 6,853 proteins and evaluated by receiver operating characteristic analysis. The generalizability of plasma protein predictors was evaluated in an external Alzheimer's Disease Research Center cohort of 369 NHW and AA study participants diagnosed as AD dementia or CU.

**Result:**

Thirty‐six proteins were selected as predictors. Proteins selected by machine learning were enriched for processes previously associated with AD including synaptic dysfunction, inflammation, vascular dysfunction, and amyloid processing. The 36 plasma proteins alone achieved an area under the curve (AUC) of 0.94 to classify AD dementia in the discovery cohort, representing a 22% improvement over a model including age and sex. The 36 proteins were validated in an external cohort also achieving AUC=0.94, a 35% improvement over age and sex.

**Conclusion:**

Biomarker discovery combining untargeted proteomics and machine learning identified a set of plasma proteins that could serve as a novel and highly accurate diagnostic plasma biomarker panel for AD. Work is underway to assess the association of the predictive panel with *p*‐tau217, medical comorbidities, and social/structural determinants of health.

